# Characterization and Genomic Analyses of *Pseudomonas aeruginosa* Podovirus TC6: Establishment of Genus *Pa11virus*

**DOI:** 10.3389/fmicb.2018.02561

**Published:** 2018-10-25

**Authors:** Chaofei Tang, Chuanjiang Deng, Yi Zhang, Cong Xiao, Jing Wang, Xiancai Rao, Fuquan Hu, Shuguang Lu

**Affiliations:** Department of Microbiology, Army Medical University, Chongqing, China

**Keywords:** *Pseudomonas aeruginosa*, phage TC6, *Pa11virus*, structural protein, comparative genomic analysis, podovirus

## Abstract

Phages have attracted a renewed interest as alternative to chemical antibiotics. Although the number of phages is 10-fold higher than that of bacteria, the number of genomically characterized phages is far less than that of bacteria. In this study, phage TC6, a novel lytic virus of *Pseudomonas aeruginosa*, was isolated and characterized. TC6 consists of an icosahedral head with a diameter of approximately 54 nm and a short tail with a length of about 17 nm, which are characteristics of the family *Podoviridae*. TC6 can lyse 86 out of 233 clinically isolated *P*. *aeruginosa* strains, thus showing application potentials for phage therapy. The linear double-stranded genomic DNA of TC6 consisted of 49796 base pairs and was predicted to contain 71 protein-coding genes. A total of 11 TC6 structural proteins were identified by mass spectrometry. Comparative analysis revealed that the *P. aeruginosa* phages TC6, O4, PA11, and IME180 shared high similarity at DNA sequence and proteome levels, among which PA11 was the first phage discovered and published. Meanwhile, these phages contain 54 core genes and have very close phylogenetic relationships, which distinguish them from other known phage genera. We therefore proposed that these four phages can be classified as *Pa11virus*, comprising a new phage genus of *Podoviridae* that infects *Pseudomonas* spp. The results of this work promoted our understanding of phage biology, classification, and diversity.

## Introduction

Bacteriophages (phages) are natural components of all ecosystems, and the estimated numbers of phages are approximately 10-fold higher than that of bacteria (Breitbart and Rohwer, 2005). Phages have been exploited by scientists to understand basic biological mechanisms, such as genetic recombination, horizontal gene transfer (HGT), molecular biology, and evolution (Henry and Debarbieux, 2012). As natural killers of bacteria, phages have been used to treat human bacterial infectious diseases at the early stage of discovery (Chanishvili, 2012). Considering that the problem of drug resistance has become increasingly serious, both the fundamental research and therapeutic application of phages are re-evaluated (Cisek et al., 2017).

As a non-fermentative Gram-negative bacteria, *Pseudomonas aeruginosa* are widely distributed in soil, sewage, medical institutions, and other environments (Stover et al., 2000). It is also an important opportunistic pathogen, which is the main Gram-negative bacteria causing nosocomial infections; infection caused by this pathogen can be fatal (Hilker et al., 2015; Crull et al., 2018). *P*. *aeruginosa* has a strong ability to form biofilm and possesses intrinsic drug resistance (Hauser, 2009). The emerging of multidrug-resistant (MDR) and pan-drug-resistant *P. aeruginosa* strains makes the treatment of *P. aeruginosa* infection very difficult (Ang et al., 2016).

The study of *P. aeruginosa* phages plays an increasingly promising role in combating this notorious pathogen. The use of phages to challenge clinically isolated MDR *P. aeruginosa* strains and biofilms *in vivo* and *in vitro* has continued for many years, and some of the results are very promising (Alemayehu et al., 2012; Krylov, 2014; Pires et al., 2015; Verbeken et al., 2016). However, phage therapy still faces multiple challenges. The main obstacles are that phage’s host spectrum is excessively narrow, bacteria have several pathways to resist phage predation (Labrie et al., 2010), and our understanding of phages is limited and shallow.

As of July 20, 2018, 2294 phages (2203 infecting bacteria and 91 infecting archaea) have been completely sequenced based on the data from NCBI Viral genome browser^1^. A total of 155 are sequenced phages infecting *Pseudomonas* spp. (including 132 *P. aeruginosa* strains), among which 93 are lytic phages, 34 are temperate phages, and 28 are not yet determined. Out of the lytic phages of *P*. *aeruginosa*, 41% belong to the family *Myoviridae*, 38% belong to the family *Podoviridae*, 20% belong to the family *Siphoviridae*, and approximately 1% cannot be classified at present (Ceyssens and Lavigne, 2010). The variation of the genome size of the phages in each family is large. The genome size distribution of the phages in the family *Myoviridae* is in the range of 64.1–309.2 kb, while the phage genomes of the family *Podoviridae* and *Siphoviridae* are distributed between 41.6–74.9 and 34.5–61.1 kb, respectively. The tailed phages of *Pseudomonas* spp. can be divided into 11 genera (Shen et al., 2016). However, many *Pseudomonas* phages are currently unassigned or unclassified. Given the difficulties in the implementation of phage therapy in medical practice related to the insufficient number and diversity of lytic phages that are active against *P*. *aeruginosa* (Krylov, 2014), the characterization and classification of novel *P. aeruginosa* phages will not only be important in understanding the interactions between *P*. *aeruginosa* and its phages but will also aid in the development of novel approaches against this notorious pathogen.

## Materials and Methods

### Bacterial Strains and Culture Conditions

*Pseudomonas aeruginosa* PA1 was originally isolated from a patient with respiratory tract infection (Lu et al., 2013, 2015; Li et al., 2016). A total of 233 clinically isolated *P*. *aeruginosa* strains were previously collected from 7 different Chinese Hospitals (Yang et al., 2016). All the above-mentioned *P*. *aeruginosa* strains are stored in our laboratory. Bacteria were grown in Luria–Bertani (LB) liquid medium with shaking or plated onto LB medium containing 1.5% (w/v) agar. The incubation temperature for all bacterial strains was 37°C.

### Phage Isolation, Propagation, and Purification

Phage TC6 was isolated from the sewage of Southwest Hospital (Chongqing, China) on the basis of a standard lambda phage isolation protocol (Sambrook and Russell, 2006). Briefly, 1000 mL sewage samples were centrifuged at 10000 *g* for 10 min. Subsequently, the supernatant was filtered through a 0.22 μm filter. Then, 3 mL PA1 culture at the log phase was added to the supernatant and cultured at 37°C for 12 h. The culture was filtered again through a 0.22 μm filter to remove the bacteria. The supernatant was serially diluted and spotted on the bacterial lawn according to the double-agar layer method. After incubation at 37°C overnight, single plaque was separated, and its phages were obtained and added to a culture of PA1 at the log phase. After incubation at 37°C for 6 h, a few drops of chloroform were added to the culture. Then, the culture was centrifuged for 5 min at 10000 *g* and filtered through a 0.22 μm filter. The resulting phage lysate was stored at 4°C for further analysis. TC6 crude phage suspensions were concentrated and purified by PEG8000 precipitation according to the method described previously (Govind et al., 2006). The purified TC6 particles were further purified using CsCl gradient ultracentrifugation (Casas and Rohwer, 2007). The optimum multiplicity of infectivity (MOI) of TC6 was observed using the method reported before (Huang et al., 2013).

### One-Step Growth Curve

The one-step growth curve of TC6 was performed as described previously (Lu et al., 2013; Latino et al., 2014). Briefly, the early logarithmic growth phase cultures of *P. aeruginosa* PA1 (OD_600_ = 0.2) were continuously diluted 10 times. Bacteria cells were calculated by CFU plate counts. Phage samples were added to 3 mL PA1 cultures (OD_600_ = 0.1) at a MOI of 10:1. After incubation at 37°C for 15 min to allow the adsorption of phages, the mixture was then centrifuged for 30 s at 13000 *g*. The supernatant containing any un-adsorbed phages was removed by washing twice with LB medium. Pellets were re-suspended in 3 mL LB and the cultures were then grown at 37°C, 180 rpm shaking. Ten microliters of samples were taken every 10 min, and the titer of TC6 particles was determined by the double-layer agar plate method. The burst time and burst size were calculated based on the one-step growth curve.

### Host Range Analysis

Host range analysis was performed as described previously (Yang et al., 2016). Phage sensitivity of 233 clinically isolated *P*. *aeruginosa* strains was determined by dot plaque assay. Briefly, 5 mL of molten 0.7% LB agar containing 100 μL of each test bacterial culture was overlaid on 1.5% LB agar plates. Subsequently, 1 μL of TC6 (∼10^10^ PFU/mL) was spotted on the soft agar. Phage resistance/susceptibility was determined by the formation of clear plaques after overnight culture at 37°C.

### Transmission Electron Microscopy (TEM)

Transmission Electron Microscopy analysis was performed as described previously (Shen et al., 2016). Briefly, filtered phage lysates (∼10^10^ PFU/mL) were placed on copper grids to allow adsorption for 10 min, then negatively stained with 2% phosphotungstic acid (pH of 4.5) for 1 min and subsequently air dried. Phage particles were observed using TECNAI 10 electron microscope (Philips, Netherlands) at a voltage of 80 kV and with a magnification of 200000. Images were acquired digitally with a camera (Gatan, Model 785) inside the microscope.

### Phage Genome Sequencing

DNA extraction and purification were performed as described previously (Lu et al., 2013). Briefly, TC6 particles purified by CsCl gradient ultracentrifugation were digested by proteinase K (50 μg mL^-1^) at 56°C for 1 h to release phage DNA. Then, the mixture was purified with phenol/chloroform/isoamyl alcohol (25:24:1) at 5000 *g* for 10 min. The aqueous layer was extracted again with chloroform at 5000 *g* for 10 min. Isopropanol was used to precipitate DNA, and the precipitated DNA was collected and washed with ethanol. The obtained TC6 DNA was suspended in TE buffer (pH of 8.0) for further use. Genomic DNA sequencing was carried out at Shanghai Biotechnology Corporation (Shanghai, China) by using PacBio sequencing technology (Rothberg et al., 2011; Gochez et al., 2018). Briefly, about 10 μg purified TC6 genomic DNA was subjected to sequencing by using PacBio RS (Pacific Biosciences, Menlo Park, CA, United States). SMRTbell template libraries with DNA fragments of 5 kb were prepared. TC6 genomic DNA was fragmented using Covaris microTUBE (ThermoFisher Scientific, MA, United States) and then purified by AMPure PB Beads^2^. Sequencing was then performed using one SMRT cells and zero-mode waveguide (ZMW) signals were obtained. After raw reads were filtered (filter criteria: minimum polymerase read quality: 0.75; minimum polymerase read length: 3,500), subreads were filtered (filter criteria: minimum subread length: 3,500); then read quality was calculated as 0.86. Approximately 78% of the filtered subreads were TC6 related. Filtered subreads were *de novo* assembled using the SPAdes v. 3.5.0 software package (Bankevich et al., 2012) with default parameters, and single contig with an average sequence coverage of 5700-fold revealed.

### Sequence Analysis and Genome Annotation

The software package DNAStar (Rosseel et al., 2012) was used to analyze the general features of the TC6 genome sequence. TC6 genes were predicted using RAST^3^ (Aziz et al., 2008; McNair et al., 2018). DNA sequences and protein sequences were scanned for homologs by using BLAST (Boratyn et al., 2013; Singh and Raghava, 2016). The software tRNAscan-SE^4^ was used to predict tRNA genes (Lowe and Chan, 2016). The circular presentation of the TC6 genome was performed using BLAST ring image generator (BRIG)^5^ (Alikhan et al., 2011) and CGView^6^ (Grant and Stothard, 2008), and the results of BRIG and CGView were combined to present the genome map of TC6.

### Identification of the TC6 Structural Proteins

Identification of the TC6 structural proteins was performed as described previously (Lu et al., 2013). Briefly, purified TC6 particles were heat denaturized and loaded onto a 10% (w/v) polyacrylamide gel to visualize TC6 structural proteins. Proteins were stained with Coomassie Brilliant Blue R250 dye and washed with methanol/acetic acid/water. Then, distinct protein bands were excised from the gel for high-performance liquid chromatography-mass spectrometry (HPLC-MS) analysis by using an HPLC-CHIP-MS/MS ION TRAP 6330 system (Agilent, Santa Clara, CA, United States). Data generated by HPLC-MS analysis were processed using Agilent Spectrum Mill proteomics software (Rev A.03.02.060; Agilent, Santa Clara, CA, United States) to allocate each protein band to its corresponding gene.

### Comparative Genomic Analysis

The Circos diagram of nucleotide sequence alignment of *Pseudomonas* phages TC6, O4 (Li et al., 2010; Zhang et al., 2018), PA11 (Kwan et al., 2006), IME180 (GenBank accession no. MF788075) and PaP2 (GenBank accession no. NC_005884) were constructed using the software Circoletto^7^ (Darzentas, 2010). TC6 genome sequence was used as reference. The tBlastX analysis of phage genomes was performed using EasyFig^8^ (Sullivan et al., 2011). The major capsid protein sequences of podoviruses infecting *Pseudomonas* spp. were downloaded from GenBank (Benson et al., 2018). Multiple sequence alignments of major capsid protein sequences were conducted using ClustalW (Hung et al., 2015) with default parameters, and phylogenetic tree was constructed by MEGA 7.0.14^9^ (Kumar et al., 2016) with the neighbor-joining method (Telles et al., 2018). Core genes were analyzed using CoreGenes3.5 (Zafar et al., 2002; Mahadevan et al., 2009). Venn diagrams were made by using an online tool^10^.

## Results and Discussion

### Morphology of TC6

**FIGURE 1:**
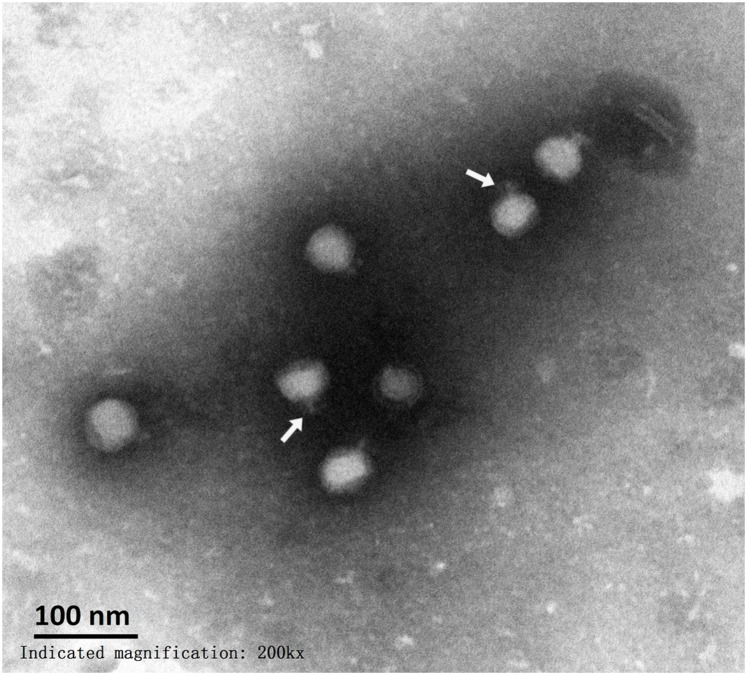
Electron micrograph of TC6 phage particles. Sample was stained with phosphotungstate. Scale bar represents 100 nm. White arrows indicate short tails.

Among the sequenced phages, approximately 90% belonged to the order *Caudovirales* (Ceyssens and Lavigne, 2010). Tailed phages have double-stranded DNA and can be divided into three different families according to different tail structures, as follows: *Podoviridae*, short and inflexible tail; *Myoviridae*, long and contractile tail; and *Siphoviridae*, long, flexible, but noncontractile tail (Grose and Casjens, 2014; Buttner et al., 2016). TEM analysis indicated that TC6 head structure was an icosahedron with a diameter of approximately 54 nm (Figure 1). Short and inflexible tail had a length of approximately 17 nm. The morphological characteristics of TC6 suggested that it should be assigned to *Podoviridae* family. TC6 had a structure and dimensions identical to that of *Pseudomonas* phage O4, which was isolated from the sea water of the coastal seashore of China Bohai inner sea, and it was also classified as a member of the *Podoviridae* family (Li et al., 2010).

### Biological Characteristics of TC6

**FIGURE 2:**
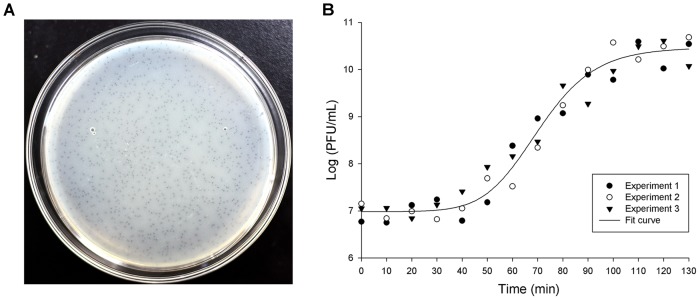
Plaques and one-step growth curve of TC6. **(A)** TC6 plaques on double agar plate. Phages were incubated at 37°C for 12 h. **(B)** One-step growth curve of phage TC6. Experiment was repeated thrice with duplicate samples at a MOI of 10:1.

Lytic phages of *P. aeruginosa* can be isolated from a variety of environments, and approximately 56% of phages are derived from hospital sewage and wastewater of wastewater treatment plants (Ceyssens and Lavigne, 2010). Separating valuable phages was more feasible for hospital sewage than for the wastewater of wastewater treatment plants because it contains many pathogenic and drug-resistant bacteria. TC6 was isolated from hospital sewage by using *P*. *aeruginosa* PA1 as the host bacterium (Li et al., 2016). After being cultured overnight (∼12 h) on double agar plate with 0.7% agar for upper LB broth, TC6 formed pin-sized plaques (∼0.5 mm in diameter) with haloes (Figure 2A). The plaque morphology of TC6 is identical to that of O4 (Li et al., 2010). In rich liquid medium, TC6 can reach high titers (∼10^10^ PFU/mL). TC6 particles are resistant to chloroform and stable for storage at 4°C. The optimum MOI of TC6 was 10:1 (i.e., phages:hosts). The one-step growth curve of TC6 showed that its latent period is approximately 40 min, and its burst period is approximately 60 min (Figure 2B). The average burst size of TC6 was estimated to be 95, that is, one host bacterium can produce approximately 95 TC6 progenies. The efficient replication of TC6 progenies suggested that TC6 was a lytic phage. A total of 233 clinically isolated *P*. *aeruginosa* strains (Yang et al., 2016) were challenged by TC6, and 86 strains can be lysed, thereby indicating potential application value of TC6 in phage therapy (Nobrega et al., 2015; Rossitto et al., 2018).

### Genomic Identification of TC6

TC6 genome is a linear dsDNA molecule containing 49796 base pairs (bp) with a G + C content of 45.06%, which is significantly less than that of its host PA1 (66.35%) (Supplementary Table S1). No intact tRNA genes were predicted in the TC6 genome. A total of 71 protein coding genes were predicted, and only 5.69% of the genome were noncoding regions (Supplementary Table S1). BlastP analysis revealed that 64 TC6 proteins had homologs in other viruses and/or bacteria, while only 38 proteins were functionally identified. A large proportion of the TC6 proteins were assigned as hypothetical proteins, which is common among phage populations (Kwan et al., 2006; Li et al., 2010; Shen et al., 2016). Numerous functionally unknown phage proteins formed the so-called viral dark matter, which are largely involved in the adaptation to host-specific conditions and the formation of tail fibers and may represent valuable information of molecular tools for biotechnological or antimicrobial applications (Filee et al., 2005; Cazares et al., 2014; Hatfull, 2015; De Smet et al., 2017).

**FIGURE 3:**
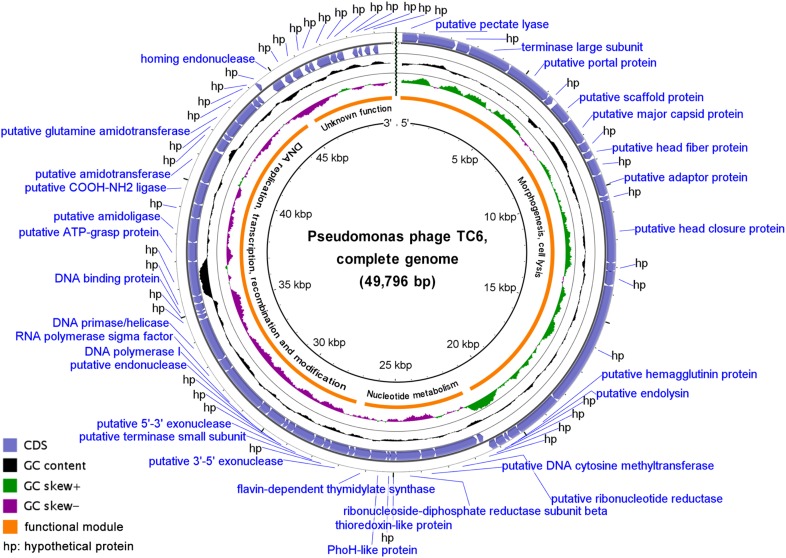
Circular presentation of the TC6 genome. The products of predicted genes were indicated in the outermost region. The outermost ring denotes genes on the plus strand, followed by rings that depict genes on the minus strand, GC content (black), GC skew (purple/green), functional modules, and position scales.

The detailed annotation and organization of the TC6 genome is illustrated in Figure 3. TC6 genome can be divided into four functional modules, of which one functionally unknown module situated near the 3′ ends of the TC6 genome, and 12 small genes with unknown functions clustered in this module (Figure 3). The gene *TC6_018* encoded a putative hemagglutinin (HA) protein of 592 aa, which is homologous to streptococcal hemagglutinin protein of *Pseudomonas* phage GP100 genome assembly, plasmid: I (GenBank accession no. LT968167) with an amino acid sequence coverage of 100% and an identity of 39%. As the major surface glycoprotein of influenza viruses, HA is a toxic protein that can agglutinate red blood cells (Byrd-Leotis et al., 2015). The putative HA protein of TC6 should be further investigated because phage-generated toxic proteins must be extensively considered during the practice of phage therapy (Watts, 2017). No homologs of phage integrases, excisionases, repressors, or transposases were predicted in the TC6 genome, thereby supporting our conclusion that TC6 is a lytic phage.

### Analysis of the Structural Proteins of TC6

**FIGURE 4:**
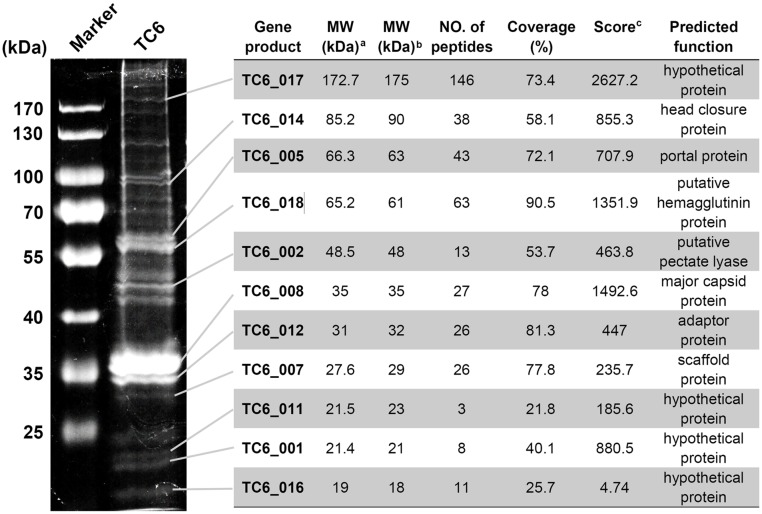
Identification of TC6 structural proteins. Proteins were visualized by SDS-PAGE analysis in a 10% (w/v) gel. The detailed results of HPLC-MS analysis were indicated on the right side of the figure. ^a^MW value was theoretically calculated. ^b^MW value was experimentally estimated. ^c^Distinct summed MS/MS search score.

A tailed phage particle was mainly made up of a series of structural proteins and a genomic DNA molecule inside its head. Genes encoding TC6 structural proteins clustered in a functional module that is situated near the 5′ ends of the TC6 genome (Figure 3). To identify the structural proteins of TC6 experimentally, we used sodium dodecyl sulfate-polyacrylamide gel electrophoresis (SDS-PAGE) to visualize the main structural proteins in the denatured gel (Figure 4). Approximately 19 protein bands with molecular weights ranging from 18 to 190 kDa were resolved. Protein bands were excised for HPLC-MS analysis. A total of 11 protein bands were identified as the products of different TC6 genes, leaving several protein bands unassigned (Figure 4). This result may be caused by the experimental errors during HPLC-MS. The predominant band was the major capsid protein (TC6_008, ∼35 kDa), which was the same as the predicted result. The major capsid protein of TC6 shared similar amino acid sequences and molecular weights to those of the *P*. *aeruginosa* phages O4 (Li et al., 2010; Zhang et al., 2018), PA11 (Kwan et al., 2006), and IME180 (GenBank accession no. MF788075), thereby suggesting a close relationship among these phages. Four hypothetical proteins, including TC6_001, TC6_011, TC6_016, and TC6_017, were separated by SDS-PAGE (Figure 4), thereby indicating that they are actually structural proteins. The gene *TC6_002* encoded a putative pectate lyase of 464 aa, which was identified as a structural component of the TC6 virion (Figure 4). TC6_002 possessed a conserved domain homologous to pectate_lyase_3, which belongs to the pectate lyase superfamily that possesses a beta helical structure. This family is most closely related to glycosyl hydrolase family 28. As a structural protein, TC6_002 may participate in host cell wall lysis to promote DNA injection.

### Whole Genome Comparison Analyses of TC6

**FIGURE 5:**
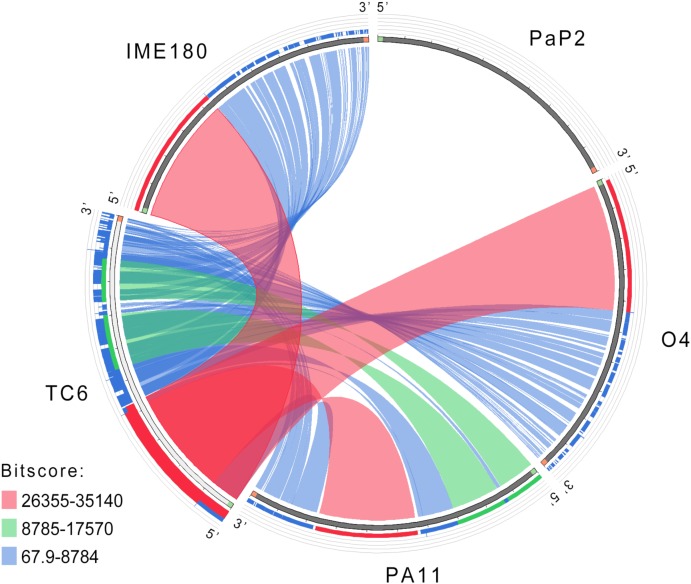
Circos diagram of nucleotide sequence alignment. Ribbons represent the similarity levels among the linked phage genome sequences. Different ranges of BLAST alignment bitscores are shown with distinct colors.

TC6 genome sequence was searched as a query in the nucleotide database of National Center for Biotechnology Information (NCBI^11^; Bethesda, MD, United States) (Sharma et al., 2018). The result showed that the genome sequences of O4 (Li et al., 2010; Zhang et al., 2018), PA11 (Kwan et al., 2006), and IME180 (GenBank accession no. MF788075) shared similar query coverage above 85% and identity above 96% with TC6 genome (Supplementary Table S2). Similar phages were isolated in different areas around the world, including Asia and North America, thereby suggesting complex evolutionary relationships among these phages. The genomic similarities of TC6, O4, PA11, IME180, and the control podovirus PaP2 (GenBank accession no. NC_005884) are illustrated in Figure 5. The whole genome sequences of TC6, O4, PA11, and IME180 showed remarkable similarities, thereby indicating that these phages may descend from a common ancestral phage. PaP2 was also isolated and sequenced in our laboratory, but it did not share genome sequence similarity with TC6. The genomic regions situated near the 3′ ends of these similar genomes showed lower similarities than the regions situated near the 5′ ends (Figure 5). This result indicated that the 3′ ends regions of these similar genomes may be highly variable during phage evolution, and some of them can be exchanged by HGT (Hafstrom et al., 2011).

**FIGURE 6:**
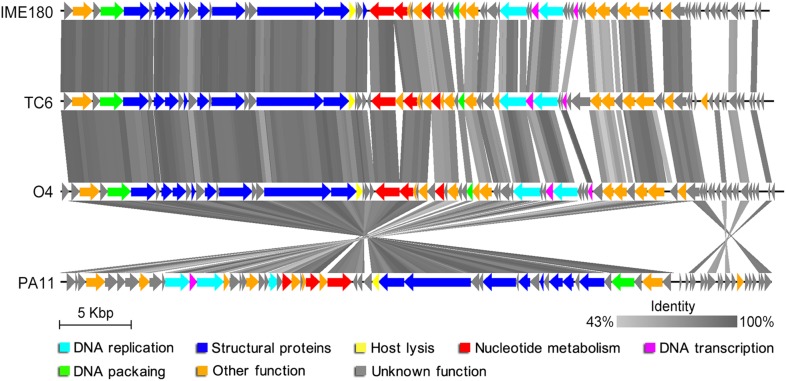
Visualized tBlastX comparison of phage genomes. Arrowheads denote genes, and the identity cut-off is set at 43%. Other function indicates putative pectate lyase, putative DNA cytosine methyltransferase, thioredoxin-like protein, PhoH-like protein, DUF3310 domain-containing protein, putative 3′–5′ exonuclease, putative 5′–3′ exonuclease, putative endonuclease, putative ATP-grasp protein, putative amidoligase, putative COOH-NH2 ligase, putative amidotransferase, putative glutamine amidotransferase, homing endonuclease, or putative serine/threonine protein phosphatase.

tBlastX analysis provided the translated comparisons of genome sequences; it revealed that TC6, O4, PA11, and IME180 showed remarkable similarities at the protein level (Figure 6). The identity of regions near the 3′ ends of these genomes were relatively lower than the average level (Figure 6), and the 3′ end regions were functionally unknown modules with small proteins of unknown functions. This phenomenon may have resulted from phage mutation accumulation to adapt to the changes in their living environment.

### Establishment of Genus *Pa11virus*

**FIGURE 7:**
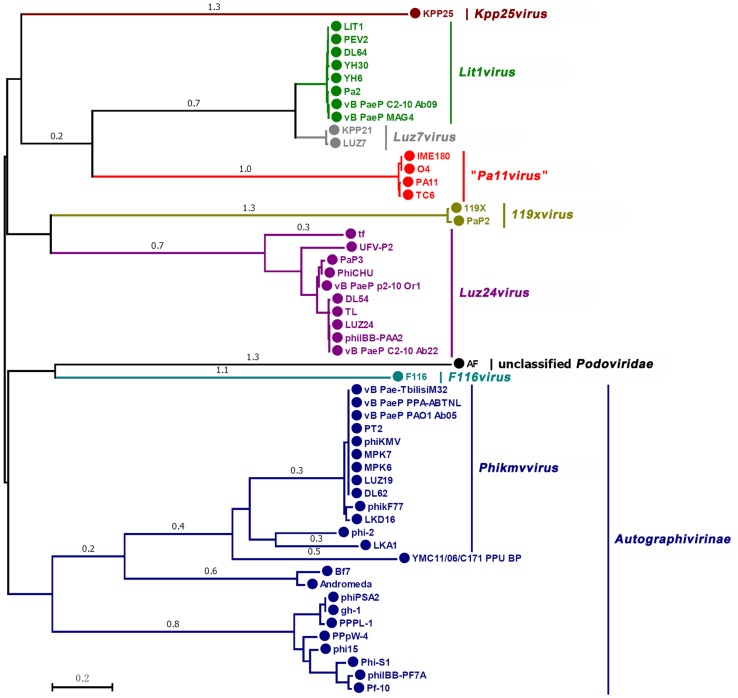
Phylogenetic relationships of *Podoviridae* phages infecting *Pseudomonas* spp. Major capsid proteins and neighbor-joining method were used to construct the phylogenetic tree. Different clades are marked with different colors. The name of each clade is identical with the GenBank database, except that the *Pa11virus* (marked in red) was proposed in this work. The scale length of relative evolution distance is 0.2.

Taxonomically similar phages can be clustered by their major capsid proteins because they are considered to have similar head structural components (Bamford et al., 2005). Therefore, to determine the taxonomical location of TC6, we performed phylogenetic analysis on the basis on the major capsid proteins. As of July 20, 2018, 349 phages of the family *Podoviridae* have been completely sequenced based on the data from the GenBank database, including 53 phages infected *Pseudomonas* spp. Among 53 *Pseudomonas* phages, 38 of which infected *P*. *aeruginosa*, 5 infected *P*. *fluorescens*, 5 infected *P*. *putida*, 3 infected *P*. *syringae*, 1 infected *P*. *tolaasii*, and 1 infected *P*. *plecoglossicida*. We selected 53 *Pseudomonas* phages to analyze the phylogenetic relationships on the basis of their major capsid protein sequences, and a phylogenetic tree was constructed (Figure 7). The result showed that phages of the same genus were clustered into one clade, thereby dividing the phylogenetic tree into several distinct clades (Figure 7). TC6, O4, PA11, and IME180 were closely clustered, distinguishing them from other *Pseudomonas* podoviruses. This finding further indicated that these four phages were descendants of the same ancestor.

Phages TC6, O4, PA11, and IME180 shared remarkable similarity (Figures 5, 6), of which average nucleotide identity (ANI) reached to 97.25% (Supplementary Table S2). These phages also had very close phylogenetic relationships, which differentiated them from other known phage genera (Figure 7). Therefore, these phages should be grouped as a new phage genus. We named this novel phage genus according to the publication date and genomic sequence release date of the first phage discovered, that is, “PA11-like phages” (the publication date and genomic sequence release date of PA11 were 2006 and 2009, respectively). We then renamed “PA11-like phages” as “*Pa11virus*” according to the suggestions of Krupovic et al. (2016), as shown in Figure 7.

**FIGURE 8:**
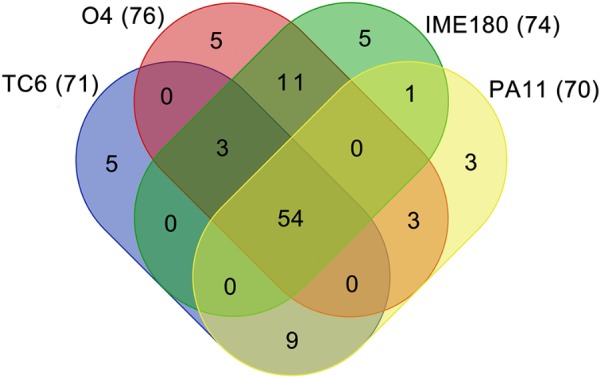
Venn diagram of genomes of *Pa11virus*. The results of core genes analysis are shown. The number of proteins were indicated in parenthesis.

The genus *Pa11virus* had a mean genome size of 49860 bp (SD = 390 bp), a mean G + C% of 44.76% (SD = 0.19%), and share 54 core genes (Figure 8). A total of 45 accessory genes were predicted, and 18 of which are unique (only belonging to one of the four phages). In the future, increasing number of newly characterized phages will be assigned to the genus *Pa11virus*, thereby improving our understanding of phage biology and diversity.

## Conclusion

This work focused on the morphological, genomic, proteomic, and comparative analyses of *P. aeruginosa* phage TC6, which is a lytic phage that belongs to the family *Podoviridae*. TC6 can lyse 86 out of 233 clinically isolated *P*. *aeruginosa* strains, thereby showing potential application for phage therapy. A total of 4 hypothetical proteins were determined as structural proteins, which improved the understanding on phage proteome. *P*. *aeruginosa* phages TC6, O4, PA11, and IME180 share remarkable similarity and have very close phylogenetic relationships, which differentiate them from other known phage genera. Accordingly, we proposed that they should be classified as a new phage genus of *Podoviridae* that infects *Pseudomonas* spp., which was named as *Pa11virus* according to the publication date of the first phage that was discovered (PA11). The results of this work will contribute to the diversity and classification of tailed phages.

## Data Availability Statement

The complete genome sequence and annotations of TC6 have been deposited in GenBank under the accession number MG676466.

## Author Contributions

SL and CT conceived and designed the experiments. CT, CD, YZ, and CX performed the experiments. SL and JW analyzed the data. JW, XR, and FH contributed reagents, materials, and analysis tools. SL wrote the paper.

## Conflict of Interest Statement

The authors declare that the research was conducted in the absence of any commercial or financial relationships that could be construed as a potential conflict of interest.

## Footnotes

1 https://www.ncbi.nlm.nih.gov/genomes/GenomesGroup.cgi?opt=virus&taxid=10239

2 http://www.pacb.com/products-and-services/consumables/pacbio-rs-ii-consumables/sample-and-template-preparation-kits/

3 http://rast.nmpdr.org/

4 http://trna.ucsc.edu/tRNAscan-SE/

5 http://brig.sourceforge.net/

6 http://stothard.afns.ualberta.ca/cgview_server/

7 http://tools.bat.infspire.org/circoletto/

8 http://mjsull.github.io/Easyfig/

9 http://www.megasoftware.net/

10 http://bioinformatics.psb.ugent.be/webtools/Venn/

11 http://www.ncbi.nlm.nih.gov/
