# Supplementary material for: Characterization and Genomic Analyses of Pseudomonas aeruginosa Podovirus TC6: Establishment of Genus Pa11virus
Source: Front Microbiol. 2018 Oct 25;9:2561. doi: 10.3389/fmicb.2018.02561 (PMC6209634; doi:10.3389/fmicb.2018.02561)
Supplement: Supplementary file 1 [file Table_1.DOCX]

Supplementary Material

Characterization and Genomic Analyses of *Pseudomonas aeruginosa* Podovirus TC6: Establishment of PA11-like Phage Genus

Chaofei Tang, Chuanjiang Deng, Yi Zhang, Cong Xiao, Jing Wang, Xiancai Rao, Fuquan Hu, Shuguang Lu*

*** Correspondence:** Shuguang Lu: shulang88@126.com

**Supplementary Table S1**. General features of the TC6 genome.

| **Features** | **TC6 genome** |
| --- | --- |
| Genome size | 49,796 bp |
| G+C content (G+C content host) | 45.06% (66.35%) |
| No. of predicted genes (proteins) | 71 (71) |
| Average length of protein-coding sequences | 661 (bp) |
| % of the genome with non-coding regions | 5.69% |
| No. of proteins without homologs | 7 |
| No. of proteins with predicted functions | 31 |
| tRNAs | No intact tRNA predicted |

**Supplementary Table S2**. Comparison of phages with BlastN query coverage above 80% of the TC6 genome.

| **Phage** | **Isolated**  **place** | **Accession** | **Genome size (bp)** | **% GC** | **Proteins (n)** | **tRNAs (n)** | **BlastN score** | **Query coverage (%)** | **Identity (%)** | **Ref.** |
| --- | --- | --- | --- | --- | --- | --- | --- | --- | --- | --- |
| TC6 | Chongqing, China | MG676466.1 | 49,796 | 45.06 | 71 | - | 91956 | 100 | 100 | This work |
| IME180 | Beijing, China | MF788075.1 | 49,494 | 44.62 | 74 | - | 60692 | 85 | 96 | - |
| O4 | Tianjin, China | KU535860.1 | 50,509 | 44.57 | 76 | tRNA-Arg | 61933 | 86 | 96 | ([Li et al., 2010](#_ENREF_2);[Zhang et al., 2018](#_ENREF_3)) |
| PA11 | Canada | NC_007808.1 | 49,639 | 44.78 | 70 | - | 81861 | 95 | 97 | ([Kwan et al., 2006](#_ENREF_1)) |

**References**

Kwan, T., Liu, J., Dubow, M., Gros, P., and Pelletier, J. (2006). Comparative genomic analysis of 18 Pseudomonas aeruginosa bacteriophages. *J Bacteriol* 188**,** 1184-1187.

Li, L., Yang, H., Lin, S., and Jia, S. (2010). Classification of 17 newly isolated virulent bacteriophages of Pseudomonas aeruginosa. *Can J Microbiol* 56**,** 925-933.

Zhang, F., Huang, K., Yang, X., Sun, L., You, J., Pan, X., Cui, X., and Yang, H. (2018). Characterization of a novel lytic podovirus O4 of Pseudomonas aeruginosa. *Arch Virol*.
